# Few Layer Graphene Does Not Affect Cellular Homeostasis of Mouse Macrophages

**DOI:** 10.3390/nano10020228

**Published:** 2020-01-28

**Authors:** Sowmya Malanagahalli, Diane Murera, Cristina Martín, Hazel Lin, Nadége Wadier, Hélène Dumortier, Ester Vázquez, Alberto Bianco

**Affiliations:** 1 CNRS, UPR3572, Immunology, Immunopathology and Therapeutic Chemistry, University of Strasbourg, ISIS, 67000 Strasbourg, France; sowmya92003@gmail.com (S.M.); d.murera@gmail.com (D.M.); cristina.martin.jimenez89@gmail.com (C.M.); rhlin@ibmc-cnrs.unistra.fr (H.L.); n.wadier@ibmc-cnrs.unistra.fr (N.W.); h.dumortier@ibmc-cnrs.unistra.fr (H.D.); 2 Instituto Regional de Investigación Científica Aplicada (IRICA), Universidad de Castilla-La Mancha, Avda Camilo Jose Cela, 13071 Ciudad Real, Spain; Ester.Vazquez@uclm.es; 3 Departamento de Química Orgánica, Facultad de Ciencias y Tecnologías Químicas, Universidad de Castilla-La Mancha, 13071 Ciudad Real, Spain

**Keywords:** graphene, carbon nanomaterials, autophagy, primary immune cells, bone marrow derived macrophages

## Abstract

Graphene-related materials (GRMs) are widely used in various applications due to their unique properties. A growing number of reports describe the impact of different carbon nanomaterials, including graphene oxide (GO), reduced GO (rGO), and carbon nanotubes (CNT), on immune cells, but there is still a very limited number of studies on graphene. In this work, we investigated the biological responses of few layer graphene (FLG) on mouse macrophages (bone marrow derived macrophages, BMDMs), which are part of the first line of defense in innate immunity. In particular, our paper describes our findings of short-term FLG treatment in BMDMs with a focus on observing material internalization and changes in general cell morphology. Subsequent investigation of cytotoxicity parameters showed that increasing doses of FLG did not hamper the viability of cells and did not trigger inflammatory responses. Basal level induced autophagic activity sufficed to maintain the cellular homeostasis of FLG treated cells. Our results shed light on the impact of FLG on primary macrophages and show that FLG does not elicit immunological responses leading to cell death.

## 1. Introduction

Isolated and characterized for the first time in 2004, graphene is an atomic monolayer of carbon atoms arranged into a hexagonal graphitic structure [1,2]. Since then, graphene and graphene-related materials (GRMs) have gained considerable attention in different fields and applications, especially in the biomedical domain [3,4]. Due to their unique properties, GRMs have found uses in water filtration systems, touch screens, medical sensors, energy storage capacitors and drug delivery [2]. Graphene research has in particular been significantly expanded to biomedical applications, which include tissue engineering, molecular imaging, antibacterial papers as well as carriers for anticancer drugs [5]. We recently highlighted why a careful characterization of GRMs is mandatory to understand the relationship between the physicochemical properties and the biological effects of these materials [6]. In the overall safety assessment of 2D materials on health and environment, one of the essential aspects is to look at the interaction of graphene and GRMs with immune cells and evaluate their responses. Studies on the whole genome expression of human peripheral blood mononuclear cells (PBMCs) from healthy donors after exposure to small and large GO (graphene oxide) showed differential expression of genes associated with different immunological pathways, while the viability of cells was not significantly affected by both small and large GO [7]. This study showed how graphene materials of different dimensions are able to regulate the immune response, looking at differential gene expression and activation patterns of molecular expression.

Many studies on the biocompatibility of GRMs have been reported mainly on the mouse macrophage cell line RAW 264.7. Wang et al. observed that RAW 264.7 cells incubated with GO functionalized with polyethylene glycol (PEG) and polyethylenimine (PEI) were 90% viable at low concentrations and that GO-PEG could trigger an inflammatory response eliciting IL-6 secretion [8]. In another study, rGO (reduced GO) microfibers inhibited cell proliferation without affecting the viability of macrophages. Inflammatory cytokine secretion showed the polarization of cells to M2 type macrophages as indicated by decreased TNF-α production and an increase in IL-6 cytokine secretion after 48 h [9]. Recently, the autophagic pathway has been considered as a key process involved in cell death and in survival mechanisms of immune cells exposed to nanomaterials. It has also been alluded to in accounting for the eventual fate of cell internalized nanomaterials [10]. Autophagy is a conserved intracellular process that delivers cytoplasmic contents to lysosomes for degradation based on the formation of double-membrane autophagosomes [10]. Cells tend to regulate autophagy, which is usually present at a basal level, but they also induce autophagy to break down parts of its own proteins or organelles to stay alive under pathophysiological conditions [11]. Autophagy is frequently activated in many immune cells, and nowadays it is widely studied to better understand the interaction of nanomaterials with mammalian cells, and even with respect to whole organs [10]. Within the class of GRMs, GO has been found to induce autophagy in cells such as macrophages and in the colon cancer cell line CT26, simultaneously activating toll-like receptors TLR 4 and TLR 9 [12,13]. Similarly, CNTs (carbon nanotubes) internalized by peritoneal macrophages induced autophagosome formation, decreased autophagic degradation and lysosomal impairment [14]. As such, we attempted to investigate the impact of this important cellular process on graphene material treatment in macrophages.

A certain number of studies on the potential biological effects of graphene materials have appeared in recent years [4,5,6]. Within the different graphene materials, FLG (few-layer graphene) is gaining in importance in fields like nanomedicine, thanks to its production in bulk quantities and its colloidal properties in biological media [15]. Even fewer papers tackle the convoluted issue of material uptake, which involves detailed and laborious procedures such as the gold standard method of transmission electron microscopy (TEM) to track intracellular material. Other studies on cellular internalization of FLG involved the preparation of radioactive labeled-sheets [16,17,18] but studying material uptake remains a very challenging task.

Indeed, this was likely neglected in the past due to issues related to the dispersibility of graphene in physiological media typically used in cell culture. Leon et al. were able to produce FLG by mechanochemical exfoliation of graphite, which is well dispersible in cell culture media (CCM) [19]. Russier et al. demonstrated that FLG is able to selectively kill tumor cells from myelomonocytic leukemia patients [20]. Recently, it has been reported that FLG can induce oxidative stress and endoplasmic-mediated autophagy in RAW264.7 macrophages [21]. In primary T and B cells, in contrast, our group has found that FLG does not impact viability and activation, nor autophagic activity [22].

It must be noted that various cells harbor different responses to FLG, and nanomaterials in general, and it is therefore pertinent to conduct separate studies independently. To expand the studies on other primary immune cells in particular, we decided to evaluate the effect of FLG on primary macrophages, derived from mouse bone marrow cells, with respect to their immunological and autophagic functions. For this purpose, we looked at the survival and activation of macrophages both in terms of upregulation of surface markers and secretion of pro-inflammatory cytokines. The impact of FLG on autophagic activity was also studied together with the internalization of the material within the cells, using TEM. In summary, our results show that FLG was internalized mainly into phagosomes by bone marrow derived macrophages and neither affected cell viability and homeostasis, nor induced inflammatory response.

## 2. Materials and Methods

### 2.1. Preparation of FLG

A mechanochemical exfoliation method using a ball milling technique in the presence of melamine as the exfoliating agent was used to obtain FLG. A mixture of graphite and melamine was ball milled at 100 rpm for 30 min and the resulting solid mixture was dispersed in water and briefly sonicated, obtaining a dark suspension. Melamine was then washed away by dialysis, and some precipitate, consisting in poorly exfoliated graphite, was eliminated from the liquid fraction after stabilization for five days. The final FLG water dispersions were lyophilized and the final graphene powder was characterized and used in the cell experiments following the dispersion in culture media [19]. The characterization of FLG by elemental analysis, TEM and Raman spectroscopy, was previously published by Murera et al. [22]. Briefly, the lateral size of the nanomaterial ranges from 100 nm to 1600 nm, the Raman average spectrum of FLG is composed of the typical graphene Raman bands: G peak, D peak, and 2D band, which are centered at ~1580 cm^−1^, ~1350 cm^−1^, and ~2680 cm^−1^, respectively, and elemental analysis showed that the sample is mainly composed of carbon, with a maximum of 0.93 ppm of melamine traces. The number of layers in FLG was acquired via an established formula, which relates the different intensities of G and 2D bands of FLG and graphite, respectively. According to this calculation, our FLG is composed of ~3 layers [23].

### 2.2. Isolation and Differentiation of Bone Marrow Cells to BMDMs

All experiments were carried out in conformity with the 2010/63/UE European animal bioethics legislation (French decree #2013-118 – 1st February 2013) and were approved by the Regional Ethics Committee of Strasbourg (CREMEAS) and by the French Ministry of Higher Education and Research (APAFIS#3280-2015121815099907 v2). Bone marrow cells (BMCs) were isolated from femurs from 9 to 18-week-old C57BI6/J mice. Femurs were trimmed at both ends and BMCs were flushed out using RPMI 1640 medium supplemented with 10% FBS, 10 mM HEPES buffer (4-(2-hydroxyethyl)-1-piperazineethanesulfonic acid), 10 µg/mL gentamycin sulfate and 0.05 mM β-mercaptoethanol (complete RPMI medium) into Petri dishes. After one wash with medium, bone marrow cells were treated with ACK lysis buffer (154.4 mM ammonium chloride, 10 mM potassium bicarbonate and 97.3 µM EDTA tetrasodium salt) for 4 min to lyse red blood cells (RBCs). Cells were then washed twice using RPMI 1640 supplemented medium. BMCs were dispersed in RPMI 1640 supplemented medium containing 30% L929-conditioned medium (i.e., supernatant from the culture of M-CSF-producing L929 cells), seeded into Petri dishes and incubated at 37 °C in CO_2_ incubator for 3 days. Afterward, the old medium was removed and replenished with a new medium for the next 3–4 days. Cells were detached upon attaining confluence and used for further experiments.

### 2.3. Addition of FLG to BMDMs

In 24-well plates, 0.5 × 10^6^ or 1 × 10^6^ cells were added and allowed to adhere for 4 h or overnight. Once the cells have adhered (confirmed by optical microscopy), they were washed once with the RPMI-supplemented medium to remove non-adherent cells. An FLG stock solution of 100 µg/mL was prepared by dispersing pre-weighed FLG in RPMI-supplemented medium containing 30% L929-conditioned medium. Before adding the FLG to the cells, FLG was sonicated for 30–60 s at intervals of 20 s in a water bath. The concentrations of FLG used were 3, 10, 30, and 100 µg/mL and the incubation time was 24 h.

### 2.4. Transmission Electron Microscopy (TEM)

For TEM imaging, BMDMs were cultured in 24-well plates as previously described at a density of 1 × 10^6^ cells per well and allowed to adhere before exposure to 100 µg/mL FLG for 24 h along with control untreated cells. After incubation, the cells were washed with cacodylate buffer twice and then fixed in 2.5% glutaraldehyde in cacodylate buffer at 4 °C overnight. Following overnight fixation, the cells were rinsed thrice with cacodylate buffer alone. Later, the cells were post-fixed with 0.5% osmium tetroxide for 30 min at room temperature and were washed thrice by MilliQ water. Cells were then dehydrated through a series of ethanol baths: 1 × 25% ethanol for 10 min, 1 × 50% ethanol for 15 min, 1 × 70% ethanol for 15 min, 1 × 95% ethanol for 10 min, and 3 × 100% ethanol for 15 min. Following dehydration, the cells were soaked in a 1:1 ratio of 100% ethanol and Epon^™^ overnight at 4 °C. The next day, the cells were rinsed (2 × 4 h) with Epon^™^. After soaking, the final inclusion of Epon^™^ into the cells was done by polymerizing Epon^™^ at 40 °C for 30 min then incubating at 60 °C for 48 h. Afterward, the polymerized blocks were removed and sliced into ultrathin sections using a diamond knife attached to an ultramicrotome cutter (Leica). The ultrathin sections were then collected on copper grids coated with Butvar^®^ B-98 and stained with 1% uranyl acetate for 30 min followed with lead citrate staining for 2 min. Grids were then examined by TEM (Hitachi H600, Krefeld, Germany). Control cells without FLG underwent the same procedure.

### 2.5. Flow Cytometer Analyses

Viability and activation of 24 h FLG-treated BMDMs were assessed using flow cytometry (Gallios, Beckman Coulter, Villepinte, France). Control (untreated), FLG-treated and LPS (lipopolysaccharide of *E. coli* 0111:B4, Sigma Aldrich, Darmstadt, Germany-treated cells were detached by washing once with cold PBS and incubated with 2% fetal bovine serum (FBS) and 0.5 mM EDTA in PBS (FACS Buffer) for 10 min at 37 °C. After incubation, the cells were detached from the plates by pipetting. Then they were washed twice (with 2% FBS in PBS). After washing, the cells were stained with an antibody mix at 4 °C for 20 min. The antibodies used were specific for CD11b (PerCP/Cy5.5 anti-mouse/human, Clone M1/70, #101228 from Biolegend, San Diego, CA, USA), F4/80 (APC, anti-mouse, Clone BM8, #123116 from Biolegend), F4/80 (PE anti-mouse, Clone BM8, #123110 from Biolegend), to identify mouse macrophage populations. For the activation of BMDMs, antibodies specific for CD80 (FITC Hamster anti-mouse, Clone 16-10A1, #553768, from BD Pharmingen) and CD86 (PE rat anti-mouse, Clone GL1, #553692, from BD Pharmingen, Le Pont de Claix, France) were used. The viability of cells was analyzed by staining with Fixable Viability Dye (FVD-eFluor 780, #65-0865-14, from eBioscience, San Diego, CA, USA). After staining, cells were washed twice by centrifuging at 1700 rpm for 2 min. Then cells were dispersed in FACS buffer and analyzed on the flow cytometer. The relevant controls were set up as shown in Figure S1A,B.

### 2.6. ELISA

Secretion of the pro-inflammatory cytokines IL-6, IL-1β and TNF-α by macrophages left untreated, LPS-treated or treated with different concentrations of FLG (i.e., 3, 10, 30, and 100 µg/mL), were assayed using a double sandwich ELISA according to the manufacturer’s instructions. In short, polyvinyl microtiter 96-well plates (Falcon) were coated overnight at 4 °C with 50 µL/well of purified rat anti-mouse IL-6 (0.5 mg/mL, BD Pharmingen #554400), rabbit anti-mouse TNF-α (0.5 mg/mL, BD Pharmingen #557516) and IL-1β (1 mg/mL, BD Biosciences, Le Pont de Claix, France), #51_26661E), diluted in coating buffer (Carbonate/bicarbonate buffer 0.05 M, pH 9.6). After washing with PBS containing 0.05% Tween (PBS-T), a saturation step was performed by adding RPMI complete medium (100 µL/well) for 1 h at 37 °C. After washing with PBS-T three times, 50 µL of culture supernatants from untreated, FLG-treated and LPS-treated macrophages were added in the respective wells for 2 h at 37 °C, along with mouse recombinant IL-6 (BD Pharmingen #554582,), recombinant TNF-α (BD Pharmingen #554589) and recombinant IL-1β (BD opti EIA kit, #51_26666E,), individually as standards. The plates were then washed five times with PBS-T. The supernatant was spun down to remove all FLG and cells, before the ELISA procedure. For secondary antibodies, biotin anti-mouse IL-6 (0.5 mg/mL, BD Pharmingen #554402), biotin anti-mouse TNF-α (0.5 mg/mL, BD Pharmingen #557432) and biotin anti-mouse IL-1β (1 mg/mL, BD Biosciences, #51_26662E) were incubated for 1 h at room temperature. Then, the plates were washed five times with PBS-T, and an equal volume of streptavidin conjugated to horseradish peroxidase (diluted 1/400) was added per well. After 30 min of incubation at room temperature, the plates were washed with PBS-T and twice with MilliQ water. The presence of cytokines in the tested supernatants was visualized by adding tetramethylbenzidine (TMB) in the presence of H_2_O_2_. The resulting absorbance was measured at 450 nm after stopping the reaction with 1 N HCl, after 15 min.

### 2.7. Western Blots

To evaluate autophagic activity, whole-cell proteins were extracted from macrophages treated with low (10 µg/mL) and high (100 µg/mL) concentrations of FLG for 24 h along with untreated and LPS-treated cells using Laemmli buffer (Tris-HCl 125 mM pH 6.8, 2% (*w*/*v*) sodium dodecyl sulfate (SDS); 10% (*v*/*v*) glycerol; 5% (*v*/*v*) β-mercaptoethanol). Cell lysates were separated on a 4–20% gradient gel (Biorad, Schiltigheim, France) and then transferred onto a polyvinylidene difluoride (PVDF) membrane. Cell membranes were blocked with PBS containing 0.1% (*v*/*v*) Tween 20 (PBS-T) and 5% (*w*/*w*) non-fat dry milk for 1 h and incubated 50 min at 4 °C with a 1 µg/mL anti-LC3 antibody in PBS-T containing 5% non-fat dry milk. The antibodies used for western immunoblotting were specific for the LC3 autophagic marker (MBL, clone 51-11, #M115-3) and the β-actin loading control (C4 mouse monoclonal antibody, Santa Cruz Biotechnology, #47778). After washing with PBS-T, the membranes were incubated for 30 min at room temperature with goat anti-mouse IgG antibody (Southern Biotech, Birmingham, Alabama, #1030-05) conjugated to horseradish peroxidase (HRP). The signal was detected using enhanced chemiluminescence detection reagents (Immobilon Western, Merck Millipore, Darmstadt, Germany, #WBKLS0500) and visualized on radiographic film in a Kodak processor (#M35-M X-OMAT, Rochester, NY, USA).

### 2.8. Statistical Analysis

The experiments were conducted at least three times. The data were processed by GraphPad Prism 6. The results are expressed as mean ± standard deviations (SD). The mean values of each treated group were statistically compared with untreated samples using One-way ANOVA followed by Bonferroni’s test, with *p* < 0.05 considered as statistically significant.

## 3. Results and Discussion

### 3.1. Internalization of FLG by BMDMs

**Figure 1:**
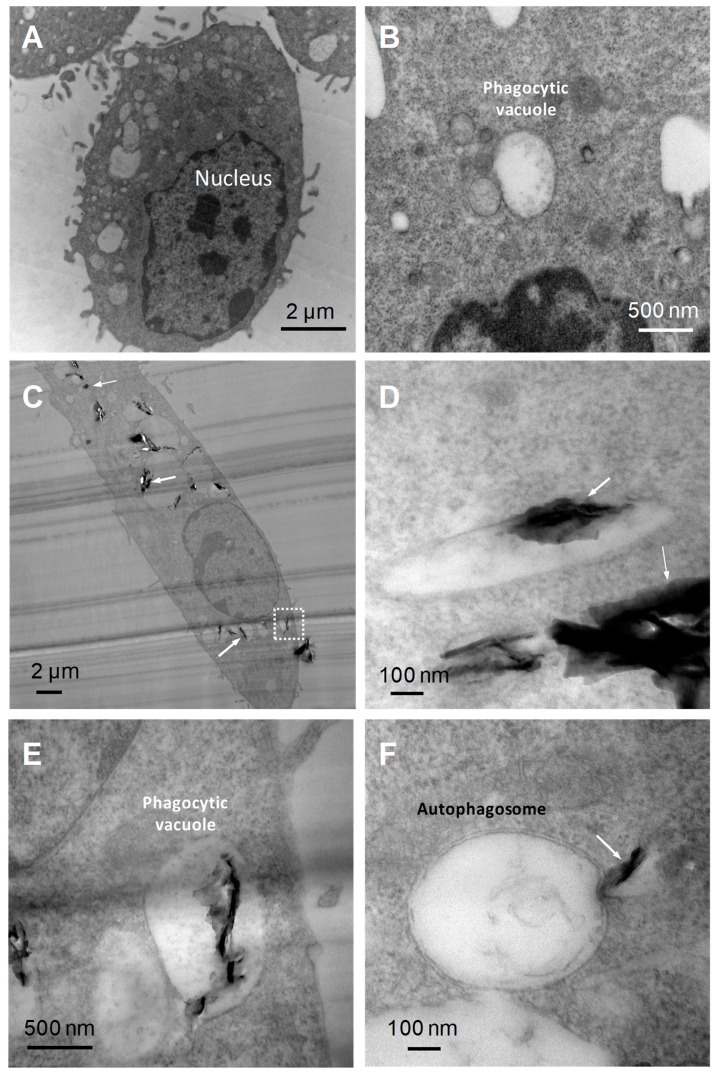
TEM images of BMDMs (bone marrow-derived macrophages). (**A**) Untreated BMDMs. (**B**) Magnified image of a phagocytic vacuole in untreated BMDMs. (**C**) BMDMs after 24 h incubation with high-dose FLG (few-layer graphene) (100 µg/mL) showing the nucleus and FLG internalized within the vacuoles and in the cytoplasm. (**D**) Magnified image of FLG into a phagocytic vacuole and into the cytoplasm. (**E**) Magnified image of phagocytic vacuole (white dotted box in panel (**C**) showing internalized FLG in a vacuole. (**F**) Double-membrane autophagosome with FLG in process of penetration.

We initially observed the internalization of FLG within BMDMs, hence we compared the TEM images of BMDMs interacting with high dose (100 µg/mL) FLG for 24 h with untreated control cells. The TEM images (Figure 1) allowed us to observe phagocytic vacuoles both in the control and FLG-treated cells. In FLG-treated cells, we could observe FLG enclosed within these phagocytic vacuoles (Figure 1C,D, indicated by thick white arrows) and as well as FLG localized in the cytoplasm (Figure 1C,D, indicated in thin white arrows), likely due to an internalization of the material via passive diffusion (e.g., FLG sheets below the white dotted box in Figure 1C, near the membrane, ‘sliding through’ cell membrane). The presence of FLG enclosed in large vacuoles (Figure 1E) indicates instead that the internalization of the material mainly occurs through phagocytosis, a characteristic of macrophages. Therefore, TEM images captured account for internalization of the FLG partly by passive diffusion and mainly by phagocytosis. These results are in agreement with previous works. For instance, Russier et al. showed phagocytic uptake and passive diffusion of GO within human and murine primary macrophages [24].

Autophagosomes, as characterized by double-membrane intracellular organelles which may or may not engulf particles, were also observed in FLG-treated cells under TEM, but not in the control untreated cells. These autophagosomes were not seen to physically engulf FLG although it seemed as if their production was triggered by the presence of material. FLG was also observed to slide into the autophagosome (Figure 1F), although no successful internalization was seen, unlike with the phagocytic vacuoles. As such, we decided to conduct further experiments to confirm if there were significant changes in the possibly relevant autophagic pathway using various assays such as the measurement of LC3 protein using western blotting.

### 3.2. Autophagic Activity

Autophagy is a process of degradation of cellular material enclosed by double-membrane structures called autophagosomes. Autophagosomes eventually fuse with lysosomes to trigger the degradation of cellular materials [25]. Autophagy is stimulated in cells upon depletion of nutrients and energy, and it has also been observed upon the encounter of cells with exogenous bodies such as bacteria, viruses, and nanomaterials [26,27]. The process of autophagy is monitored by the expression of autophagy-related proteins. Among these proteins, the most commonly used to monitor the autophagic process is the microtubule-associated light chain 3 (LC3). The conversion of the cytosolic form of LC3 (LC3-I) into membrane-associated LC3-phosphatidylethanolamine conjugate (LC3-II), which is recruited by autophagosomal membranes, leading to the induction of autophagic activity, is commonly used as a marker of autophagy.

**Figure 2:**
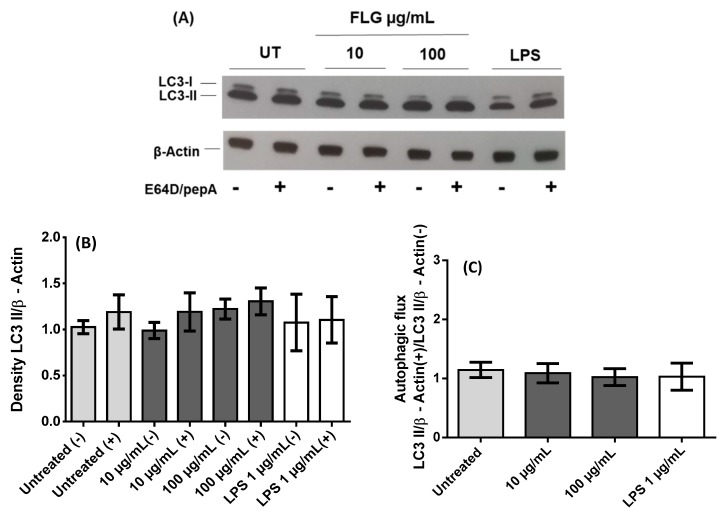
Autophagy analysis in BMDMs after 24 h interaction with FLG. (**A**) Representative Western blot of different samples as indicated for LC3 proteins along with internal loading control β-actin both in the presence (+) and the absence (−) of protease inhibitors E64D and pepstatin A (pepA). Untreated control (UT) (**B**) Relative quantification of density ratio of LC3-II to β-actin corresponding to conditions mentioned earlier. (**C**) Densitometric evaluation, using ImageJ, of the autophagic flux considering the ratio between the presence (+) and the absence (−) of E64D/pepA of the autophagic activity (LC3-II/β-actin). A One-way ANOVA followed by Bonferroni’s post-test was performed to determine the statistical differences for control untreated cells versus FLG-treated samples and LPS-treated cells (*p* > 0.05).

A growing body of literature suggests that internalized nanoparticles may undergo autophagic sequestration. This may lead to autophagy dysfunction and cause cell death, thus playing a key role in nanoparticle toxicity. To investigate how FLG influences autophagy in primary macrophages, BMDMs were incubated with low (10 µg/mL) and high (100 µg/mL) doses of graphene for 24 h. A representative immunoblot (Figure 2A) of cell lysates of untreated, low- and high-dose FLG-treated and LPS-treated conditions shows the expression of LC3-I and -II with or without lysosomal protease inhibitors (E64D and pepstatin A). The expression of LC3 proteins in low- and high- dose FLG-treated cells is similar to basal level as observed in untreated cells and LPS-treated cells [22] and was observed to be slightly higher in the presence of protease inhibitors, indicating the inhibition of the degradation of autophagosomes. The visualization of LC3 bands is, however, not enough to attest to the influence of FLG on autophagic activity induction in macrophages, hence the densitometric ratio was calculated between membrane-bound LC3-II protein and β-actin (internal loading reference). From Figure 2B it can be inferred that autophagic activity in the absence of protease inhibitors is slightly less than all conditions tested in comparison to the presence of protease inhibitors, due to inhibitory action as mentioned earlier. LPS, which showed no increase in the presence of protease inhibitors, predictably showed a very slight decrease in autophagic flux, as visualized in Figure 2C, although there was eventually no significant difference between all conditions. Autophagic flux was calculated by considering the ratio between the expression of LC3-II in the presence and absence of protease inhibitors. We can, therefore, deduce that the autophagic activity remains similar to basal level as seen in untreated cells (Figure 2C). These results are in accordance with cell viability and cellular activation both in terms of expression of activation molecules and cytokine secretion. Interestingly, the role of autophagy in the impact of graphene materials has been previously highlighted. Many papers have already alluded to autophagic dysfunction as a cause of cytotoxicity. Graphene quantum dots, another member of the graphene family, was reported to cause apoptosis and autophagy in THP-1-derived macrophages [28]. Di Cristo et al. used FLG dispersed in BSA and investigated autophagic activity in RAW 264.7 macrophages, where the authors reported an increase in autophagic activity in comparison to control cells [21]. Here, we used primary macrophages and FLG dispersed in cell culture media, which is close to the natural physiological environment, reporting an autophagic activity that is similar to basal level. Our work demonstrates instead that FLG possibly but minimally modulates autophagic activity.

### 3.3. Cell Viability

**Figure 3:**
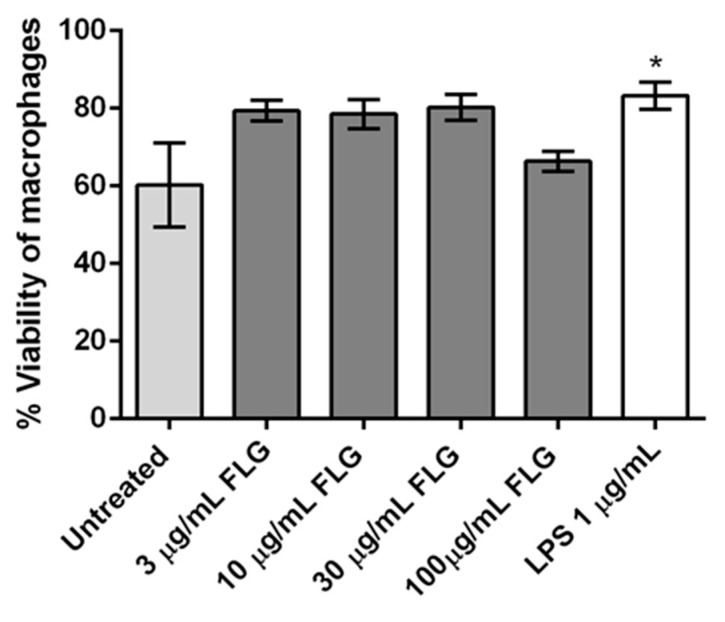
Flow cytometry analysis of cellular viability of BMDMs treated with different concentrations of FLG (3, 10, 30 and 100 µg/mL), with LPS (1 µg/mL) and left untreated, for 24 h. One-way ANOVA followed by Bonferroni’s test was performed to determine the statistical differences among samples versus control untreated cells (* *p* < 0.05).

It is equally crucial to know how FLG affects the viability of BMDMs, as macrophages are the first line of defenders in innate immunity. Viability was assessed using the viability marker FVD via flow cytometry. FVD distinguishes live and dead cells by covalently cross-linking to both extra- and intra-cellular proteins of cells with compromised membranes (Figure 3). The viability of BMDMs treated with 3, 10, and 30 µg/mL FLG seemed slightly improved compared to untreated control, as commonly observed in other experiments in our lab, although without any statistical difference. The observed behavior of the cells is consistent with earlier reports where the viability of primary embryonic bovine cells treated with rGO and murine macrophage cell line RAW 264.7 treated with FLG was not affected [21,29]. We observed increased survival of macrophages treated with 3, 10, and 30 µg/mL of FLG. This was observed in microscopic images of BMDMs after 24 h interacting with FLG, where morphological changes of proliferative cells such as attachment, elongation, and number of cell colonies were lower in untreated cells in comparison to FLG- treated cells (Figure S2). Similar proliferative behavior has been shown for osteoblastic MC3T3-E1 cells, induced by GO scaffolds [30], and in primary human macrophages exposed to pristine graphene and pristine single-walled CNTs, which all indicated no loss of viability [31]. Our data show that the viability of BMDMs is not detrimentally affected by FLG. This was further confirmed by looking at caspase-3 activity identifying apoptotic populations (Figure S3). Interestingly, viability was observed to be slightly improved in conditions such as LPS.

### 3.4. Cell Activation

**Figure 4:**
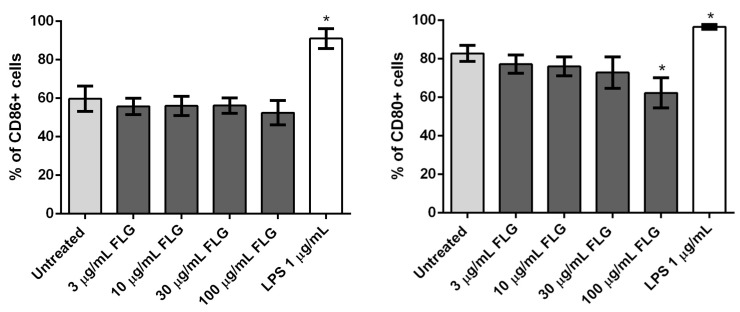
Flow cytometry analysis of CD86 (**left**) and CD80 (**right**) expression in BMDMs left untreated as a control or treated with different concentrations of FLG (3, 10, 30 and 100 µg/mL) or with LPS (1 µg/mL), for 24 h. %CD86 and %CD80 refer to the percentage of live gated cells that express CD86 or CD80 respectively. A One-way ANOVA followed by Bonferroni’s post-test was performed to determine the statistical differences for control untreated cells versus FLG-treated samples and LPS-treated cells (* *p* < 0.05).

Cell activation, in turn, is also an important parameter in checking the impact of nanomaterials in phagocytosing immune cells such as macrophages. We investigated the upregulation of surface activation markers CD80 and CD86 on BMDMs exposed to FLG [32]. CD80 and CD86 expression were measured on untreated, FLG-treated and LPS-treated cells (24 h) using flow cytometry (Figure 4). No statistically significant effect on CD86 expression was observed, though the positive control LPS increased CD86 as expected. Treatment with FLG did not affect CD80 expression either, except for a small decrease at the highest dose (Figure 4).

### 3.5. Cytokine Measurement

**Figure 5:**
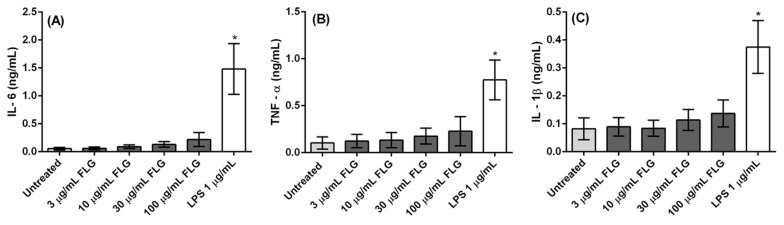
Cytokine secretion by BMDMs. (**A**) IL-6, (**B**) TNF-α, and (**C**) IL-1β levels in cell supernatant after treatment with or without FLG (3, 10, 30, 100 µg/mL), and LPS (1 µg/mL) for 24 h. A One-way ANOVA followed by Bonferroni’s post-test was performed to determine the statistical differences for control untreated cells versus FLG-treated samples and LPS-treated cells (* *p* < 0.05).

To further explore the activation profile of the macrophages following their incubation with different concentrations of FLG (3, 10, 30, and 100 µg/mL) along with untreated and LPS-treated cells, we determined the titers of pro-inflammatory cytokines (IL-6, TNFα, and IL-1β) in the culture supernatants by ELISA. As shown in Figure 5, IL-6 secretion slightly increases with FLG concentration, though not significantly when compared to untreated cells. Similarly, secretion of pro-inflammatory cytokines TNF-α and IL-1β also increases with FLG dose but again there was no significant difference in cytokine secretion profile. Notably, neutralization or decreased secretion of cytokines has little to no impact on cell survival, as observed earlier in cell viability assays [33]. The absence of significant inflammatory cytokine secretion indicates that an inflammatory response in macrophages was not elicited by FLG.

## 4. Conclusions

Our paper provides a closer look, using transmission electron microscopy, at the impact of FLG on one of the most relevant immune cells involved in uptake and interaction of foreign material – the macrophage. We subsequently investigated the response of FLG on crucial cellular parameters such as cytotoxicity, cytokine production, and autophagic processes.

We found that FLG was internalized within the cells through passive diffusion or phagocytosis. As inflammasome activation is induced by a wide range of stimuli, such as microorganisms, metabolic dysregulation as well as by nanomaterials [34], phagocytosed FLG was predicted to evoke inflammasome activity, however, in this study we did not observe any significant increase in inflammasome-dependant IL-1β production. This was accompanied by no significant secretion of other pro-inflammatory cytokines such as IL-6 and TNF-α. Altogether, this indicates that FLG did not evoke inflammatory response in primary bone marrow derived macrophages.

Moreover, this work demonstrates that the viability of primary mouse macrophages was not affected by FLG after 24 h of incubation. In addition, upregulation of the activation marker CD86 was not induced in FLG-treated cells, and we even found a slight decrease in CD80 expression. Despite the control experiments we performed reassure us regarding the validity of our results, we are fully aware of possible experimental artifacts conducting common assays such as ELISA and flow cytometry when using FLG and other graphene-related material. Indeed, the material can either increase or decrease the resultant signal at many stages of sample processing and measurement, thereby affecting the interpretation of data [35]. Of note, the slight quenching of FVD and other conjugated antibody fluorescent signal due to FLG, may be circumvented by saturating the amount of dye used.

Finally, the experiments aimed to assess the induction of autophagic activity in primary macrophages by FLG showed that both low and high doses of FLG did not affect the basal level of autophagic activity, showing that FLG does not induce any cellular stress.

Overall, our work demonstrates that a short period of interaction of FLG with primary macrophages does not induce an inflammatory response nor cell toxicity. However, more experiments are required to further investigate the different immunological pathways along with in vitro and in vivo studies to look at the mechanistic processes towards cellular homeostasis.

## Supplementary Materials

The following are available online at https://www.mdpi.com/2079-4991/10/2/228/s1, Figure S1. Control experiments for flow cytometry stainings. Control unstained BMDMs are shown in red while DMSO-treated e780-FVD-stained positive cells (Figure S1A) and CD80-FITC-stained positive cells (Figure S1B) are shown in blue. Figure S2. Optical microscopy images of untreated, low (10 µg/mL) and high (100 µg/mL) concentration of FLG and LPS (1 µg/mL)-treated BMDMs for 24 h. Figure S3. Flow cytometry analysis of viability and apoptosis of BMDMs treated with different concentrations of FLG (3, 10, 30 and 100 µg/mL), with LPS (1 µg/mL) and left untreated, for 24 h. (A) Representative dot plots, and (B) corresponding bar graph of percentages of total population of cells showing viable, early apoptotic, late apoptotic and dead population of cells.

## References

[1] Zhang B., Wei P., Zhou Z., Wei T. (2016). Interactions of Graphene with Mammalian Cells: Molecular Mechanisms and Biomedical Insights. Adv. Drug Deliv. Rev..

[2] Zhang Y., Nayak T.R., Hong H., Cai W. (2012). Graphene: A Versatile Nanoplatform for Biomedical Applications. Nanoscale.

[3] Mukherjee S.P., Bottini M., Fadeel B. (2017). Graphene and the Immune System: A Romance of Many Dimensions. Front. Immunol..

[4] Ou L., Song B., Liang H., Liu J., Feng X., Deng B., Sun T., Shao L. (2016). Toxicity of Graphene-Family Nanoparticles: A General Review of the Origins and Mechanisms. Part. Fibre Toxicol..

[5] Sanchez V.C., Jachak A., Hurt R.H., Kane A.B. (2012). Biological Interactions of Graphene-Family Nanomaterials: An Interdisciplinary Review. Chem. Res. Toxicol..

[6] Fadeel B., Bussy C., Merino S., Vázquez E., Flahaut E., Mouchet F., Evariste L., Gauthier L., Koivisto A.J., Vogel U. (2018). Safety Assessment of Graphene-Based Materials: Focus on Human Health and the Environment. ACS Nano.

[7] Orecchioni M., Jasim D.A., Pescatori M., Manetti R., Fozza C., Sgarrella F., Bedognetti D., Bianco A., Kostarelos K., Delogu L.G. (2016). Molecular and Genomic Impact of Large and Small Lateral Dimension Graphene Oxide Sheets on Human Immune Cells from Healthy Donors. Adv. Healthc. Mater..

[8] Wang B., Su X., Liang J., Yang L., Hu Q., Shan X., Wan J., Hu Z. (2018). Synthesis of Polymer-Functionalized Nanoscale Graphene Oxide with Different Surface Charge and Its Cellular Uptake, Biosafety and Immune Responses in Raw264.7 Macrophages. Mater. Sci. Eng. C.

[9] Serrano M.C., Feito M.J., González-Mayorga A., Diez-Orejas R., Matesanz M.C., Portolés M.T. (2018). Response of Macrophages and Neural Cells in Contact with Reduced Graphene Oxide Microfibers. Biomater. Sci..

[10] Wei F., Duan Y. (2019). Crosstalk between Autophagy and Nanomaterials: Internalization, Activation, Termination. Adv. Biosys..

[11] Kaur J., Debnath J. (2015). Autophagy at the Crossroads of Catabolism and Anabolism. Nat. Rev. Mol. Cell Biol..

[12] Chen G.-Y., Yang H.-J., Lu C.-H., Chao Y.-C., Hwang S.-M., Chen C.-L., Lo K.-W., Sung L.-Y., Luo W.-Y., Tuan H.-Y. (2012). Simultaneous Induction of Autophagy and Toll-like Receptor Signaling Pathways by Graphene Oxide. Biomaterials.

[13] Chen G.-Y., Chen C.-L., Tuan H.-Y., Yuan P., Li K.-C., Yang H., Hu Y.-C. (2014). Graphene Oxide Triggers Toll-like Receptors/Autophagy Responses in Vitro and Inhibits Tumor Growth in Vivo. Adv. Healthc. Mater..

[14] Wan B., Wang Z.-X., Lv Q.-Y., Dong P.-X., Zhao L.-X., Yang Y., Guo L.-H. (2013). Single-Walled Carbon Nanotubes and Graphene Oxides Induce Autophagosome Accumulation and Lysosome Impairment in Primarily Cultured Murine Peritoneal Macrophages. Toxicol. Lett..

[15] León V., González-Domínguez J.M., Fierro J.L.G., Prato M., Vázquez E. (2016). Production and stability of mechanochemically exfoliated graphene in water and culture media. Nanoscale.

[16] Mao L., Hu M., Pan B., Xie Y., Petersen E.J. (2016). Biodistribution and toxicity of radio-labeled few layer graphene in mice after intratracheal instillation. Part. Fiber Toxicol..

[17] Lu K., Dong S., Petersen E.J., Niu J., Chang X., Wang P., Lin S., Gao S., Mao L. (2017). Biological Uptake, Distribution, and Depuration of Radio-Labeled Graphene in Adult Zebrafish: Effects of Graphene Size and Natural Organic Matter. ACS Nano.

[18] Mao L., Liu C., Lu K., Su Y., Gu C., Huang Q., Petersen E.J. (2016). Exposure of few layer graphene to Limnodrilus hoffmeisteri modifies the graphene and changes its bioaccumulation by other organisms. Carbon.

[19] González-Domínguez J.M., León V., Lucío M.I., Prato M., Vázquez E. (2018). Production of ready-to-use few-layer graphene in aqueous suspensions. Nat. Protoc..

[20] Russier J., León V., Orecchioni M., Hirata E., Virdis P., Fozza C., Sgarrella F., Cuniberti G., Prato M., Vázquez E. (2017). Few-Layer Graphene Kills Selectively Tumor Cells from Myelomonocytic Leukemia Patients. Angew. Chem. Int. Ed..

[21] Cristo L.D., Carthy S.M., Paton K., Movia D., Prina-Mello A. (2018). Interplay between Oxidative Stress and Endoplasmic Reticulum Stress Mediated- Autophagy in Unfunctionalised Few-Layer Graphene-Exposed Macrophages. 2D Mater..

[22] Murera D., Malaganahalli S., Martín C., Reina G., Fauny J.-D., Dumortier H., Vázquez E., Bianco A. (2019). Few Layer Graphene Does Not Affect the Function and the Autophagic Activity of Primary Lymphocytes. Nanoscale.

[23] Paton K.R., Varrla E., Backes C., Smith R.J., Khan U., O’Neill A., Boland C., Lotya M., Istrate O.M., King P. (2014). Scalable production of large quantities of defect-free few-layer graphene by shear exfoliation in liquids. Nat. Mater..

[24] Russier J., Treossi E., Scarsi A., Perrozzi F., Dumortier H., Ottaviano L., Meneghetti M., Palermo V., Bianco A. (2013). Evidencing the Mask Effect of Graphene Oxide: A Comparative Study on Primary Human and Murine Phagocytic Cells. Nanoscale.

[25] Mihalache C.C., Simon H.-U. (2012). Autophagy Regulation in Macrophages and Neutrophils. Expt. Cell Res..

[26] Bah A., Vergne I. (2017). Macrophage Autophagy and Bacterial Infections. Front. Immunol..

[27] Li J.J., Hartono D., Ong C.-N., Bay B.-H., Yung L.-Y.L. (2010). Autophagy and Oxidative Stress Associated with Gold Nanoparticles. Biomaterials.

[28] Qin Y., Zhou Z.-W., Pan S.-T., He Z.-X., Zhang X., Qiu J.-X., Duan W., Yang T., Zhou S.-F. (2015). Graphene Quantum Dots Induce Apoptosis, Autophagy, and Inflammatory Response via P38 Mitogen-Activated Protein Kinase and Nuclear Factor-ΚB Mediated Signaling Pathways in Activated THP-1 Macrophages. Toxicology.

[29] Park S., Mohanty N., Suk J.W., Nagaraja A., An J., Piner R.D., Cai W., Dreyer D.R., Berry V., Ruoff R.S. (2010). Biocompatible, Robust Free-Standing Paper Composed of a TWEEN/Graphene Composite. Adv. Mater..

[30] Nishida E., Miyaji H., Kato A., Takita H., Iwanaga T., Momose T., Ogawa K., Murakami S., Sugaya T., Kawanami M. (2016). Graphene Oxide Scaffold Accelerates Cellular Proliferative Response and Alveolar Bone Healing of Tooth Extraction Socket. Int. J. Nanomed..

[31] McIntyre J., Verma N.K., Smith R.J., Moore C., Nerl H., McEvoy N., Berner N., McGovern I., Khan U., Lyons P. (2016). A Comparison of Catabolic Pathways Induced in Primary Macrophages by Pristine Single Walled Carbon Nanotubes and Pristine Graphene. RSC Adv..

[32] Hoshino Y., Hoshino S., Gold J.A., Raju B., Prabhakar S., Pine R., Rom W.N., Nakata K., Weiden M. (2007). Mechanisms of Polymorphonuclear Neutrophil—Mediated Induction of HIV-1 Replication in Macrophages during Pulmonary Tuberculosis. J. Infect. Dis..

[33] Nolan A., Kobayashi H., Naveed B., Kelly A., Hoshino Y., Hoshino S., Karulf M.R., Rom W.N., Weiden M.D., Gold J.A. (2009). Differential Role for CD80 and CD86 in the Regulation of the Innate Immune Response in Murine Polymicrobial Sepsis. PLoS ONE.

[34] Lebre F., Hanlon D., Boland J.B., Coleman J., Lavelle E.C. (2018). Exfoliation in Endotoxin-Free Albumin Generates Pristine Graphene with Reduced Inflammatory Properties. Adv. Biosyst..

[35] Petersen E.J., Hirsch C., Elliott J.T., Krug H.F., Aengenheister L., Arif A.T., Bogni A., Kinsner-Ovaskainen A., May S., Walser T. (2019). Cause-and-Effect Analysis as a Tool to Improve the Reproducibility of Nanobioassays: Four Case Studies. Chem. Res. Toxicol..

