# Supplementary material for: Few Layer Graphene Does Not Affect Cellular Homeostasis of Mouse Macrophages
Source: Nanomaterials (Basel). 2020 Jan 28;10(2):228. doi: 10.3390/nano10020228 (PMC7074970; doi:10.3390/nano10020228)
Supplement: Supplementary file 1 [file nanomaterials-10-00228-s001.pdf]

Supplementary Information

Few Layer Graphene Does Not Affect Cellular Homeostasis of Mouse Macrophages

Sowmya Malanagahalli [1], Diane Murera [1], Cristina Martín [1,2], Hazel Lin [1], Nadége Wadier [1], Hélène Dumortier [1], Ester Vázquez [2,3] and Alberto Bianco [1,*]

[1] CNRS, UPR3572, Immunology, Immunopathology and Therapeutic Chemistry, University of Strasbourg, ISIS, 67000 Strasbourg, France; sowmya92003@gmail.com (S.M.); d.murera@gmail.com (D.M.); cristina.martin.jimenez89@gmail.com (C.M.); rhlin@ibmc-cnrs.unistra.fr (H.L.); n.wadier@ibmc-cnrs.unistra.fr (N.W.); h.dumortier@ibmc-cnrs.unistra.fr (H.D.)

[2] Instituto Regional de Investigación Científica Aplicada (IRICA), Universidad de Castilla-La Mancha, Avda Camilo Jose Cela, 13071 Ciudad Real, Spain; Ester.Vazquez@uclm.es

[3] Departamento de Química Orgánica, Facultad de Ciencias y Tecnologías Químicas, Universidad de Castilla-La Mancha, 13071 Ciudad Real, Spain

* Correspondence: a.bianco@ibmc-cnrs.unistra.fr

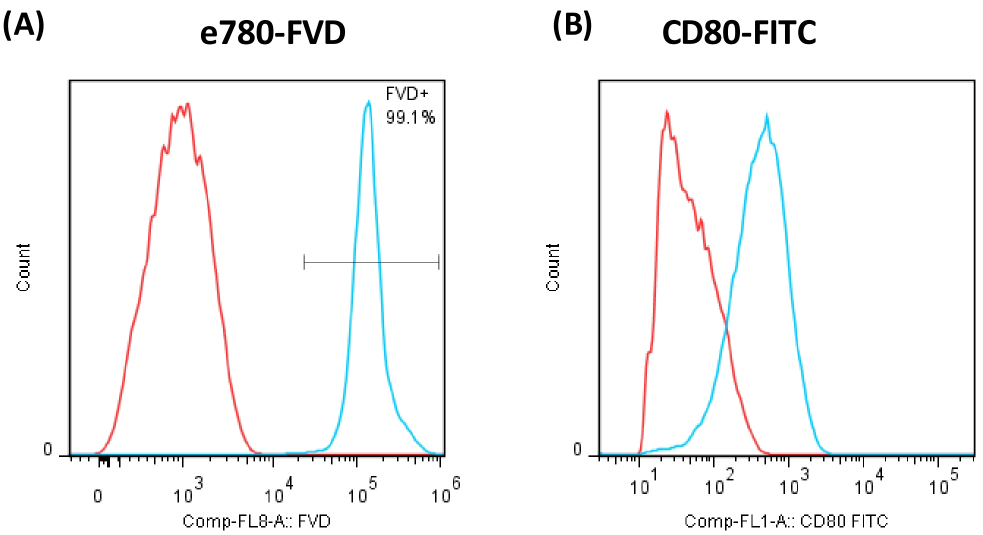

Figure. S1. Control experiments for flow cytometry stainings. Control unstained BMDMs are shown in red while DMSO-treated e780-FVD-stained positive cells (Fig S1A) and CD80-FITC-stained positive cells (Fig S1B) are shown in blue.

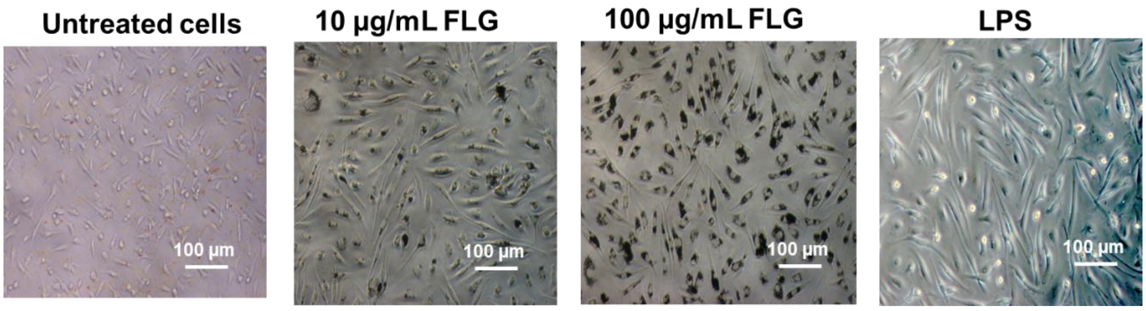

Figure. S2. Optical microscopy images of untreated, low (10 µg/mL) and high (100 µg/mL) concentration of FLG and LPS (1 µg/mL)-treated BMDMs for 24 h.

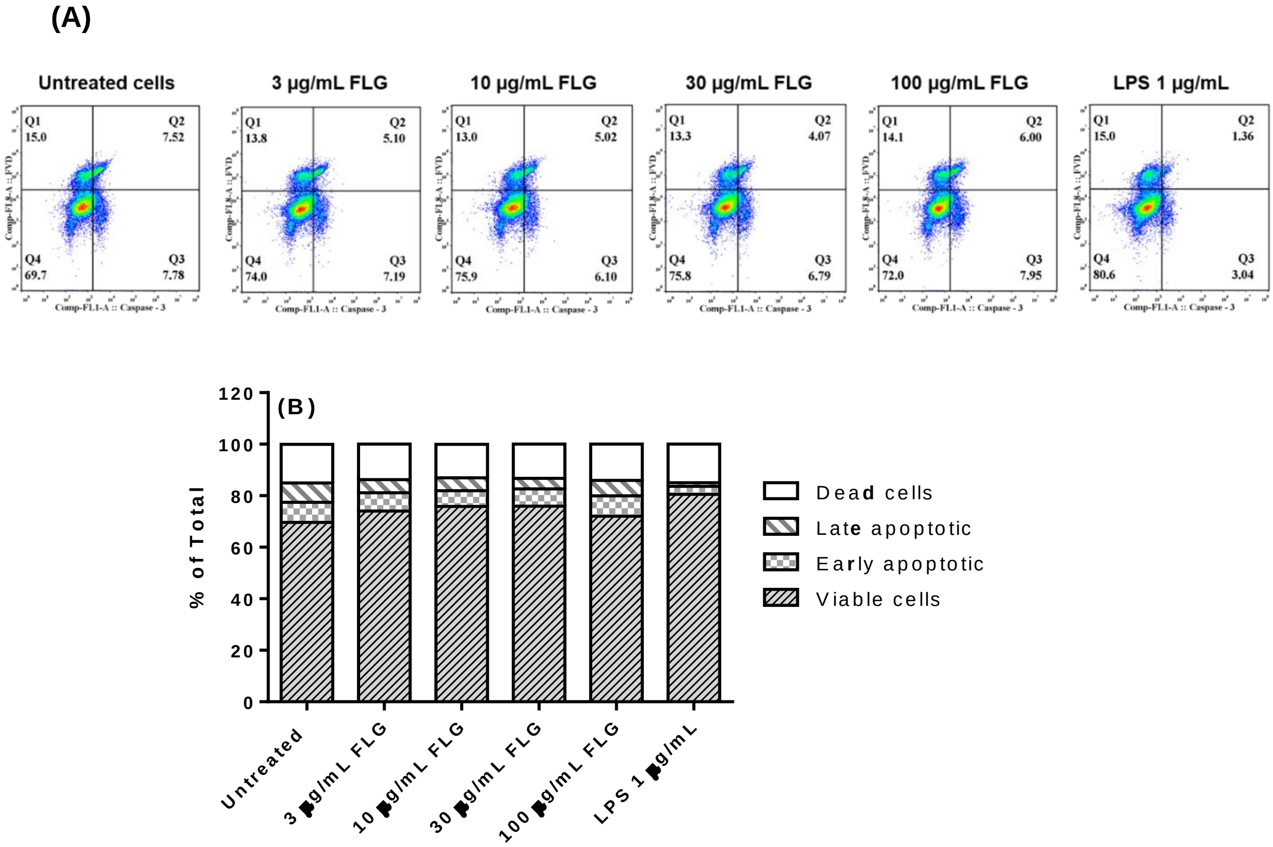

Figure. S3. Flow cytometry analysis of viability and apoptosis of BMDMs treated with different concentrations of FLG (3, 10, 30 and 100 µg/mL), with LPS (1 µg/mL) and left untreated, for 24 h. (A) Representative dot plots, and (B) corresponding bar graph of percentages of total population of cells showing viable, early apoptotic, late apoptotic and dead population of cells.
